# Association of APRI, FIB-4, and FIB-5 Scores with Threatened Miscarriage

**DOI:** 10.3390/diagnostics16121797

**Published:** 2026-06-10

**Authors:** Mehmet Efe Namlı, Hande Kurt Güven, Elif Yılmaz

**Affiliations:** Department of Obstetrics and Gynecology, Ankara Ataturk Sanatoryum Egitim Ve Arastirma Hastanesi, Ankara 06290, Turkey

**Keywords:** threatened miscarriage, abortus imminens, APRI, FIB-4, FIB-5, early pregnancy loss risk

## Abstract

**Objectives**: This study aimed to compare the aspartate aminotransferase-to-platelet ratio index (APRI), fibrosis-4 index (FIB-4), and fibrosis-5 index (FIB-5) between women with threatened miscarriage and healthy pregnant controls, and to evaluate their discriminative performance. **Methods**: This single-center retrospective case–control study included 100 women with threatened miscarriage and 100 gestational-age-matched healthy pregnant controls within the first 12 weeks of gestation. Demographic, obstetric, ultrasonographic, and laboratory data were retrieved from electronic records. APRI, FIB-4, and FIB-5 were calculated from routine laboratory parameters. Group comparisons, binary logistic regression, and ROC analyses were performed. **Results**: AST, ALT, ALP, APRI, and FIB-4 were higher, while hemoglobin, platelet count, albumin, and FIB-5 were lower in the threatened miscarriage group (all *p* < 0.001). APRI (OR = 6.937), FIB-4 (OR = 89.114), and FIB-5 (OR = 0.766) were independently associated with case status. FIB-4 showed the highest discriminative performance (AUC = 0.929), followed by APRI (AUC = 0.903) and FIB-5 (AUC = 0.761). **Conclusions**: APRI and particularly FIB-4 showed good apparent discrimination between women with threatened miscarriage and healthy controls in this retrospective dataset. However, these indices should be interpreted as exploratory laboratory-derived markers rather than disease-specific biomarkers until validated in prospective multicenter studies.

## 1. Introduction

Threatened miscarriage is defined as vaginal bleeding occurring in the presence of a viable intrauterine pregnancy while the cervix remains closed, and it is one of the most common problems encountered in early pregnancy [1,2]. This condition has been reported to occur in approximately 15–25% of pregnancies. Although the pregnancy continues in a substantial proportion of cases, some patients remain at risk of pregnancy loss [1,3]. Therefore, threatened miscarriage should be considered not merely as a transient first-trimester bleeding episode, but as a clinically meaningful condition in terms of early risk stratification and determination of follow-up strategy.

In clinical practice, threatened miscarriage is primarily evaluated using clinical findings, transvaginal ultrasonography, serial β-hCG measurements, and selected biochemical markers. However, the prognostic value of commonly studied markers such as progesterone and CA125 is limited by gestational-age-related variation, heterogeneous thresholds, and inconsistent cut-off values [3,4]. Moreover, progesterone therapy appears to benefit only selected subgroups rather than all women with threatened miscarriage [1,5]. These limitations support the investigation of inexpensive indices derived from routine laboratory tests to better characterize systemic biochemical alterations accompanying early pregnancy bleeding.

The biological basis of threatened miscarriage does not appear to be simple enough to be explained solely by hormonal changes. In recent years, evidence suggesting that the maternal inflammatory response and immune activation may accompany this condition has been steadily increasing [2,6]. Indeed, it has been reported that the proinflammatory cytokine profile is more pronounced in threatened miscarriage cases than in healthy pregnancies, and that this may be associated with a higher risk of pregnancy loss [2]. In parallel, studies have also been published showing that the systemic immune-inflammation index, neutrophil-to-lymphocyte ratio, and certain other hemogram-based inflammatory markers are increased in patients with threatened miscarriage [7]. Nevertheless, the available data are not entirely consistent. While one review demonstrated that neutrophil-to-lymphocyte ratio levels were higher in cases of pregnancy loss, a similarly consistent discriminatory value could not be established for platelet-to-lymphocyte ratio [6]. These findings suggest that the biological stress occurring in early pregnancy bleeding may be reflected in routine laboratory parameters, but that more balanced and easily applicable scores are still worth investigating for clinical use.

APRI, FIB-4, and FIB-5 were originally developed to predict liver fibrosis without requiring invasive procedures. They are low-cost and easily applicable composite scores that can be calculated from routine laboratory parameters such as AST, ALT, platelet count, age, albumin, and alkaline phosphatase [8]. The fact that APRI and FIB-4, in particular, are used for their diagnostic performance in chronic liver diseases demonstrates the importance of these indices [9]. In pregnancy, the scope of use of these scores has recently been expanding. It has been reported that APRI, FIB-4, and FIB-5 levels can differ significantly from those of control groups in hypertensive disorders of pregnancy, preeclampsia, intrahepatic cholestasis of pregnancy, and hyperemesis gravidarum [10,11,12]. This suggests that these scores may reflect not only liver injury, but also hepatocellular stress, platelet dynamics, and the systemic biological response indirectly [11,12]. Therefore, APRI, FIB-4, and FIB-5 stand out as practical candidate markers that can be evaluated in clinical conditions seen in early pregnancy without requiring additional imaging or expensive biomarkers.

This study aimed to compare APRI, FIB-4, and FIB-5 scores between women presenting with threatened miscarriage and gestational-age-matched controls, and to assess the discriminatory performance of these indices for identifying threatened miscarriage.

## 2. Materials and Methods

### 2.1. Study Design

This single-center retrospective case–control study was conducted at Ankara Atatürk Sanatorium Training and Research Hospital using data obtained from the electronic medical records of patients evaluated between 1 January and 31 December 2025.

### 2.2. Study Population and Case Definition

The study population consisted of pregnant women evaluated during the first 12 weeks of gestation. The case group included women who presented with vaginal bleeding during early pregnancy, had a closed cervical os on pelvic examination, and demonstrated a viable intrauterine pregnancy on ultrasonography, consistent with threatened miscarriage. The control group consisted of women with a confirmed viable intrauterine pregnancy who presented during the same study period without vaginal bleeding. Controls were selected from the same institution and were matched to cases according to gestational age at presentation in a 1:1 ratio, with an allowable difference of ±3 days. Maternal age was not used as a matching criterion.

The study population consisted of women receiving care at a tertiary public hospital in Ankara, Türkiye. Available demographic variables included maternal age, body mass index, employment status, education level, and income level. Ethnicity was not routinely recorded in the electronic medical records and therefore could not be analyzed as a separate variable.

### 2.3. Exclusion Criteria

Patients were excluded if they had suspected ectopic pregnancy, molar pregnancy, multiple gestation, known chronic liver disease, active hepatitis, hematologic disease, or any other clinical condition likely to substantially affect aminotransferase levels or platelet indices. Patients with incomplete laboratory data required for calculation of APRI, FIB-4, or FIB-5 scores were also excluded. In addition, cases without sufficient follow-up data to determine pregnancy outcome were not included in the final analysis.

### 2.4. Data Collection

Demographic, obstetric, clinical, and ultrasonographic data were retrieved from the institutional electronic database. The recorded variables included maternal age, gravidity, parity, gestational age at presentation, presenting symptoms, and ultrasonographic findings. Gestational age was determined according to the last menstrual period and was confirmed or revised, when necessary, using first-trimester ultrasonographic measurements documented in the medical records.

Laboratory parameters were obtained from blood samples collected at the index visit. In the case group, all laboratory measurements used for analysis were based on blood samples drawn on the same day as presentation with vaginal bleeding. In the control group, laboratory values were obtained from the blood samples collected on the day of presentation. The analyzed laboratory variables included aspartate aminotransferase (AST), alanine aminotransferase (ALT), platelet count, albumin, and alkaline phosphatase (ALP).

Medication exposure before the index visit, including progesterone or other agents that could potentially affect liver enzyme levels, was reviewed when available in the electronic medical records. However, medication use initiated before presentation or outside the institution could not be reliably verified in all patients; therefore, this variable could not be included as an adjustment factor in the main analysis.

### 2.5. Calculation of APRI, FIB-4, and FIB-5 Scores

APRI, FIB-4, and FIB-5 scores were calculated from routine laboratory parameters recorded at the index visit. The formulas used were as follows:

FIB-4 = (Age × AST [U/L])/(Platelet count [10^9^/L] × √ALT [U/L])

FIB-5 = [Albumin × 0.3 + Platelet count × 0.05] − [ALP × 0.014 + (AST/ALT ratio × 6) + 14]

APRI = [(AST/upper limit of normal AST) × 100]/Platelet count [10^9^/L]

### 2.6. Outcome Definition

The primary outcome was spontaneous pregnancy loss occurring after the index presentation. Pregnancy outcomes were determined through follow-up outpatient records, hospital documentation, and ultrasonographic reports. Only losses documented after the initial visit were considered outcome events. Among women with threatened miscarriage, cases with ongoing pregnancy beyond the first trimester or documented fetal viability during follow-up were classified as non-loss outcomes.

### 2.7. Statistical Analysis

Statistical analyses were performed using the SPSS (IBM SPSS Statistics 31) software package. Frequency tables and descriptive statistics were used in the interpretation of the findings. Parametric methods were used for measurement values that were consistent with normal distribution. In accordance with parametric methods, the “Independent Samples *t*-test” (t-table value) method was used to compare the measurement values of two independent groups. Non-parametric methods were used for measurement values that were not consistent with normal distribution. In accordance with non-parametric methods, the “Mann–Whitney U” test (Z-table value) method was used to compare the measurement values of two independent groups. “Pearson-χ^2^” cross-tables were used to examine the relationships between two qualitative variables. “Binary logistic regression: Backward LR model” was used to examine the factors affecting disease status. The “ROC” method was used to determine the APRI, FIB-4, and FIB-5 values that discriminate disease status. A *p* value of <0.05 was accepted as statistically significant.

## 3. Results

Although the groups were comparable in terms of gestational age, BMI, employment status, education level, and income level, maternal age was significantly higher in the threatened miscarriage group than in the control group (median 27.0 [5.0] vs. 25.0 [5.8] years; *p* = 0.001). Therefore, maternal age was considered a potential confounding factor, particularly for the interpretation of FIB-4.

There was no statistically significant difference between the groups in terms of body mass index and gestational week (*p* > 0.05).

There was no statistically significant relationship between group and employment status, education level, and income level (*p* > 0.05). Except for maternal age, the groups were comparable regarding the evaluated baseline characteristics (Table 1).

A statistically significant difference was found between the groups in terms of hemoglobin, platelet count, AST, ALT, albumin, ALP, APRI, FIB-4, and FIB-5 values (*p* < 0.05). It was determined that AST, ALT, ALP, APRI, and FIB-4 values of those in the study group were significantly higher than those in the control group. In addition, it was determined that hemoglobin, platelet count, albumin, and FIB-5 values of those in the control group were significantly higher than those in the study group (Table 2).

As a result of the Backward:LR logistic regression analysis performed with some parameters that may affect disease status, the optimal model is given in the table. In the current model, the APRI parameter was found to be an important parameter affecting disease status (*p* < 0.05). When the APRI value increases by 1 unit, the risk of disease will increase 6.937 times (OR = 6.937). The FIB-4 parameter was found to be an important parameter affecting disease status (*p* < 0.05). When the FIB-4 value increases by 1 unit, the risk of disease will increase 89.114 times (OR = 89.114). The FIB-5 parameter was found to be an important parameter affecting disease status (*p* < 0.05). When the FIB-5 value increases by 1 unit, the risk of disease will decrease by 13.4% (OR = 0.766) (Table 3).

**Figure 1:**
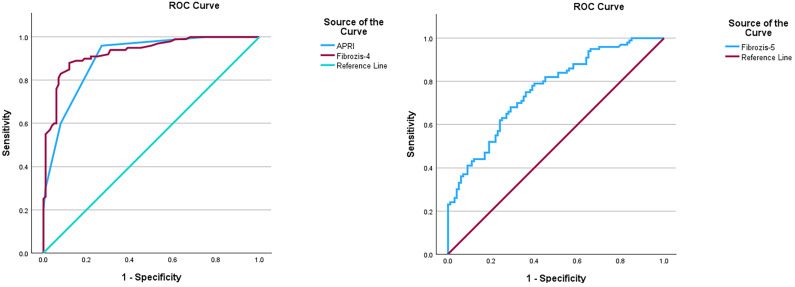
Determination of APRI, Fibrosis-4 and Fibrosis-5 values discriminating the study group by ROC.

The optimal APRI cut-off value was determined as ≥0.250 with 96.0% sensitivity and 73.0% specificity (AUC = 0.903; *p* < 0.001). The optimal FIB-4 cut-off value was determined as ≥0.635 with 88.0% sensitivity and 88.0% specificity (AUC = 0.929; *p* < 0.001). The optimal FIB-5 cut-off value was determined as ≤33.445 with 68.0% sensitivity and 71.0% specificity (AUC = 0.761; *p* < 0.001) (Table 4 and Figure 1).

## 4. Discussion

In this study, APRI and FIB-4 scores were shown to be significantly higher, while the FIB-5 score was lower, in women diagnosed with threatened miscarriage compared with healthy controls. FIB-4 showed the highest AUC in ROC analysis, followed by APRI; however, the wide confidence interval around the FIB-4 effect estimate indicates imprecision and limits direct clinical interpretation. Studies showing that inflammatory and immune balance is disrupted in threatened miscarriage and pregnancy loss also reveal that early pregnancy complications may be associated with systemic biological responses [2,13]. Therefore, it is possible that scores derived from routine laboratory parameters reflect clinically meaningful systemic changes occurring in early pregnancy [7,14]. Accordingly, APRI, FIB-4, and FIB-5 may be evaluated in this context not as direct fibrosis markers or disease-specific biomarkers, but as exploratory laboratory-derived indices reflecting the biochemical stress response accompanying threatened miscarriage.

Threatened miscarriage should not be regarded as a clinical condition limited only to uterine bleeding. In early pregnancy, disruption of the balance among maternal immune tolerance, cytokine balance, and peripheral inflammatory response may accompany the biological basis of this condition [15]. Indeed, it has been reported that the systemic immune-inflammation index changes significantly in cases of threatened miscarriage and may be used to predict pregnancy outcome. Similarly, markers derived from complete blood count such as systemic inflammation response index and systemic immune-inflammation index have been shown to reflect increased inflammatory burden [7,16]. Studies conducted in cases of threatened miscarriage using routine blood parameters and biomarkers reflecting the acute-phase response suggest that this process cannot be explained solely by local uterine events, but may be accompanied by a broader systemic biochemical response [14].

Several biochemical and hormonal markers have been investigated for the assessment of threatened miscarriage. Serum progesterone has prognostic value in early pregnancy, but its interpretation is limited by gestational-age-related variation and the lack of a universally accepted threshold [3]. Similarly, CA125 has been evaluated as an adjunctive marker for miscarriage risk, yet heterogeneity across studies and inconsistent cut-off values have limited its use as a standalone decision-making tool [4]. From a therapeutic perspective, progesterone does not appear to provide uniform benefit in all threatened miscarriage cases; its effect is more evident in selected subgroups, particularly women with early pregnancy bleeding and a previous history of miscarriage [1,5]. In this context, APRI, FIB-4, and FIB-5 should not be viewed as replacements for established clinical, ultrasonographic, or hormonal evaluation, but rather as complementary laboratory-derived indices that may reflect systemic biochemical alterations accompanying threatened miscarriage.

The present findings should also be interpreted in relation to the individual laboratory components underlying APRI, FIB-4, and FIB-5. Women with threatened miscarriage had higher AST, ALT, and ALP levels and lower hemoglobin, platelet count, and albumin levels than healthy pregnant controls. Previous studies evaluating threatened miscarriage, early pregnancy loss, and pregnancy-related inflammatory conditions have suggested that routine hematological and biochemical parameters may change in association with systemic inflammation, acute-phase response, and altered platelet dynamics [6,7,14,16]. Mild increases in AST and ALT may reflect hepatocellular or systemic biochemical stress rather than overt liver disease, while ALP may be influenced by hepatobiliary and pregnancy-related biological activity. Lower albumin may reflect inflammatory redistribution, hemodilution, nutritional factors, or altered hepatic protein synthesis. Similarly, lower hemoglobin and platelet count may be related to inflammatory activity, vascular-endothelial activation, peripheral platelet consumption, or early pregnancy-related hemodynamic changes. Therefore, the observed pattern supports the interpretation that APRI, FIB-4, and FIB-5 reflect a composite biochemical profile rather than the isolated effect of a single parameter.

From a mechanistic perspective, threatened miscarriage may involve interactions among systemic inflammation, oxidative stress, immune dysregulation, endothelial activation, and impaired maternal–fetal interface function. Inflammatory cytokine activation may affect endothelial integrity, platelet activity, hepatic acute-phase responses, and vascular homeostasis. Oxidative stress may contribute to cellular injury and altered enzyme release, whereas immune dysregulation may disturb the tolerance mechanisms required for early pregnancy maintenance. Within this framework, higher APRI and FIB-4 values may reflect the combined effect of mildly increased aminotransferases and reduced platelet count. Conversely, lower FIB-5 values may be driven by lower albumin and platelet count together with higher ALP and AST/ALT-related components. Thus, these indices should be interpreted as indirect reflections of systemic biochemical disturbance rather than markers of a single pathophysiological pathway.

It is also important to emphasize that the observed alterations in APRI and FIB-4 are unlikely to be specific to threatened miscarriage. Similar aminotransferase- and platelet-derived indices have been evaluated in other pregnancy-related complications, including hypertensive disorders of pregnancy, intrahepatic cholestasis of pregnancy, and hyperemesis gravidarum [10,11,12]. Therefore, APRI and FIB-4 should be interpreted as nonspecific indicators of systemic biochemical or inflammatory response rather than disease-specific markers for threatened miscarriage. Their potential value may lie in identifying a measurable systemic response accompanying early pregnancy bleeding, but they cannot distinguish threatened miscarriage from other inflammatory, hepatic, or obstetric conditions without appropriate clinical context.

In particular, the difference demonstrated by APRI and FIB-4 in ROC analysis suggests that indices derived from routine biochemical data may carry clinically meaningful significance in cases of threatened miscarriage. Studies focusing on serum amyloid A and maternal serum cytokine profile also support that measurable systemic biological changes accompany cases of threatened miscarriage [2,14]. In this case, our findings suggest not that APRI and FIB-4 are specific obstetric markers for threatened miscarriage, but that they may serve as auxiliary indices capable of reflecting the systemic biochemical response emerging in early pregnancy with a clinically usable level of accuracy. In contrast, the lower discriminative performance of FIB-5 indicates that this score should rather be considered a complementary indicator.

This study showed that APRI, FIB-4, and FIB-5 may serve as auxiliary indices with the potential to reflect systemic biochemical changes occurring in early pregnancy. Recent studies demonstrating that maternal serum cytokine profiles and markers reflecting the acute-phase response change in cases of threatened miscarriage support that this condition has a measurable biological counterpart [2,14]. New models attempting to predict threatened abortion risk using routine laboratory data also show that such easily accessible parameters may contribute to clinical risk stratification [17,18]. Therefore, although our findings indicate that APRI and FIB-4 in particular are meaningful in the evaluation of threatened miscarriage, these scores need to be re-tested in prospective and multicenter studies before they can be directly integrated into clinical practice.

Because the median gestational age in both groups was approximately 9 weeks, our findings may be particularly relevant to laboratory assessment during the 8th–10th weeks of gestation. Individual parameters such as platelet count, AST, ALT, ALP, hemoglobin, and albumin may provide partial information about inflammatory, hepatic, hematological, nutritional, or hemodynamic status. However, isolated interpretation of these parameters is limited because each may be influenced by several physiological and non-pregnancy-related factors. Composite indices such as APRI, FIB-4, and FIB-5 may offer a more integrated representation of these alterations by combining aminotransferase, platelet, albumin, ALP, and age-related components. Nevertheless, the present findings should be considered exploratory, and future prospective studies should evaluate whether individual parameters or composite indices measured during the 8th–10th weeks of gestation can predict subsequent pregnancy outcomes.

## 5. Strengths and Limitations

This study has several strengths. To the best of our knowledge, it is among the first studies to evaluate APRI, FIB-4, and FIB-5 in women with threatened miscarriage. The inclusion of gestational-age-matched controls from the same institution reduced variability related to pregnancy timing, and all indices were calculated from routinely available laboratory parameters obtained at the index visit. In addition, the study evaluated both between-group differences and apparent discriminative performance using ROC analysis.

Nevertheless, several limitations should be acknowledged. First, the retrospective single-center case–control design limits causal inference and may restrict generalizability. In addition, the study population was derived from a single tertiary public hospital in Ankara, Türkiye, and ethnicity was not routinely recorded in the electronic database. Therefore, population-specific demographic, environmental, or genetic factors may influence the generalizability and biomarker performance of these indices in other settings. Second, although the groups were matched for gestational age, they were not matched for maternal age; the threatened miscarriage group was significantly older than the control group. This is particularly relevant for FIB-4 because maternal age is included in its formula. Third, medication exposure before the index visit, particularly progesterone use initiated before presentation or outside the institution, could not be reliably verified in all patients. Because progesterone may influence liver enzyme levels, this represents a potential source of residual confounding. Fourth, APRI, FIB-4, and FIB-5 are formula-derived indices sharing overlapping laboratory components, particularly aminotransferase and platelet-related variables; therefore, model instability should be considered when interpreting regression results. Fifth, the wide confidence interval observed for FIB-4 indicates imprecision in the estimated effect size. Accordingly, these indices should be interpreted as exploratory laboratory-derived markers rather than validated disease-specific biomarkers for threatened miscarriage. Prospective multicenter studies with standardized medication recording and external validation are required before clinical implementation.

## 6. Conclusions

Women with threatened miscarriage had higher APRI and FIB-4 scores and lower FIB-5 scores than healthy controls. APRI and especially FIB-4 demonstrated good apparent discrimination in this retrospective case–control dataset. However, because of the retrospective design, maternal age difference between groups, possible unmeasured medication exposure, and the wide confidence interval observed for FIB-4, these scores should be interpreted as exploratory adjunctive indices rather than disease-specific biomarkers. Their diagnostic and prognostic value should be confirmed in prospective multicenter studies with standardized medication recording and external validation.

### Key Messages

Routine liver-derived indices may capture more than hepatic status in early pregnancy. In threatened miscarriage, the marked separation of FIB-4 and APRI from controls suggests a broader biochemical response that could support risk-oriented assessment without adding new tests or cost.

## Figures and Tables

**Table 1:** Comparison of socio-demographic and general characteristics according to groups.

Variable	Study (*n* = 100)	Control (*n* = 100)	Statistical Analysis *	*p*-Value
Age (year)	27.0 [5.0]	25.0 [5.8]	Z = −3.204	*p* = 0.001
BMI (kg/m^2^)	25.0 [5.0]	25.0 [5.0]	Z = −1.339	*p* = 0.181
Gestational week	8.9 [2.4]	8.9 [2.3]	Z = −0.139	*p* = 0.889
Employment status			χ^2^ = 0.021	*p* = 0.885
Yes	39 (39.0%)	40 (40.0%)		
No	61 (61.0%)	60 (60.0%)		
Education level			χ^2^ = 3.069	*p* = 0.216
Primary school/middle school	21 (21.0%)	12 (12.0%)		
High school	62 (62.0%)	67 (67.0%)		
University	17 (17.0%)	21 (21.0%)		
Income level			χ^2^ = 1.338	*p* = 0.512
Below minimum wage	15 (15.0%)	12 (12.0%)		
Minimum wage	56 (56.0%)	64 (64.0%)		
Above minimum wage	29 (29.0%)	24 (24.0%)		

* For data not showing normal distribution, “Mann–Whitney U” test (Z-table value) statistics were used in the comparison of measurement values of two independent groups and are presented as Median [IQR]. In examining the relationships between two qualitative variables, “Pearson-χ^2^” cross tables were used and are presented as *n*, (%).

**Table 2:** Comparison of some quantitative variables according to groups.

Variable	Study (*n* = 100)	Control (*n* = 100)	Statistical Analysis *	*p*-Value
Hg	12.17 ± 0.94	12.91 ± 1.09	t = −5.141	*p* < 0.001
WBC	7.3 [2.7]	7.3 [2.9]	Z = −1.774	*p* = 0.081
PLT	182.15 ± 47.89	267.79 ± 54.18	t = −11.843	*p* < 0.001
AST	24.0 [6.0]	18.0 [6.0]	Z = −8.015	*p* < 0.001
ALT	18.0 [10.0]	14.0 [9.0]	Z = −3.851	*p* < 0.001
Albumin	4.0 [0.8]	4.2 [0.7]	Z = −3.883	*p* < 0.001
ALP	68.0 [15.8]	53.5 [11.8]	Z = −9.881	*p* < 0.001
APRI	0.4 [0.2]	0.2 [0.2]	Z = −10.076	*p* < 0.001
Fib-4	0.89 [0.3]	0.44 [0.2]	Z = −10.486	*p* < 0.001
Fib-5	31.91 ± 3.73	35.81 ± 3.81	t = −7.301	*p* < 0.001

* For data showing normal distribution, “Independent Sample-t” test (t-table value) statistics were used in the comparison of measurement values of two independent groups and are presented as Mean ± standard deviation. For data not showing normal distribution, “Mann–Whitney U” test (Z-table value) statistics were used in the comparison of measurement values of two independent groups and are presented as Median [IQR].

**Table 3:** Logistic Regression model established based on disease status.

Variable	B	S.H.	Wald	df	*p*	OR	95% Confidence Interval (OR) Lower	Upper
APRI	1.937	0.280	47.914	1	<0.001	6.937	4.009	12.005
FIB-4	10.149	1.345	23.312	1	<0.001	89.114	17.514	334.190
FIB-5	−0.267	0.045	35.230	1	<0.001	0.766	0.701	0.834
Constant	−13.412	3.762	12.708	1	0.004	0.001		

CCR = 87.0% χ^2^(8) = 13.042; *p* = 0.110.

**Table 4:** Determination of APRI, Fibrosis-4 and Fibrosis-5 values discriminating the study group by ROC.

Variable	Area	Standard Error	*p*	AUC 95% C.I. Lower	Upper	Cut-Off
APRI	0.903	0.021	<0.001	0.861	0.944	≥0.250
Fibrosis-4	0.929	0.018	<0.001	0.894	0.964	≥0.635
Fibrosis-5	0.761	0.033	<0.001	0.696	0.826	≤33.445

## Data Availability

The data presented in this study are available on request from the corresponding author.

## Abbreviations

ALP: alkaline phosphatase; ALT: alanine aminotransferase; APRI: aspartate aminotransferase-to-platelet ratio index; AST: aspartate aminotransferase; AUC: area under the curve; FIB-4: fibrosis-4 index; FIB-5: fibrosis-5 index; OR: odds ratio; ROC: receiver operating characteristic.
